# Genome-wide analysis of the basic Helix-Loop-Helix (bHLH) transcription factor family in maize

**DOI:** 10.1186/s12870-018-1441-z

**Published:** 2018-10-16

**Authors:** Tingting Zhang, Wei Lv, Haisen Zhang, Lin Ma, Pinghua Li, Lei Ge, Gang Li

**Affiliations:** 1 State Key Laboratory of Crop Biology, College of Life Sciences, Shandong Agricultural University, Tai’an, 271018 China; 2 State Key Laboratory of Crop Biology, College of Agronomy, Shandong Agricultural University, Tai’an, 271018 China; 3 School of Biological Science and Technology, University of Jinan, Jinan, 250022 China

**Keywords:** Maize, Genome-wide analysis, Basic helix-loop-helix transcription factors, Expression analysis, Development

## Abstract

**Background:**

In plants, the basic helix-loop-helix (bHLH) transcription factors play key roles in diverse biological processes. Genome-wide comprehensive and systematic analyses of bHLH proteins have been well conducted in *Arabidopsis*, rice, tomato and other plant species. However, only few of bHLH family genes have been functional characterized in maize.

**Results:**

In this study, our genome-wide analysis identified 208 putative bHLH family proteins (ZmbHLH proteins) in maize (*Zea mays*). We classified these proteins into 18 subfamilies by comparing the ZmbHLHs with *Arabidopsis thaliana* bHLH proteins. Phylogenetic analysis, conserved protein motifs, and exon-intron patterns further supported the evolutionary relationships among these bHLH proteins. Genome distribution analysis found that the 208 *ZmbHLH* loci were located non-randomly on the ten maize chromosomes. Further, analysis of conserved *cis*-elements in the promoter regions, protein interaction networks, and expression patterns in roots, leaves, and seeds across developmental stages, suggested that bHLH family proteins in maize are probably involved in multiple physiological processes in plant growth and development.

**Conclusion:**

We performed a genome-wide, systematic analysis of bHLH proteins in maize. This comprehensive analysis provides a useful resource that enables further investigation of the physiological roles and molecular functions of the ZmbHLH transcription factors.

**Electronic supplementary material:**

The online version of this article (10.1186/s12870-018-1441-z) contains supplementary material, which is available to authorized users.

## Background

Maize (*Zea mays*) is an important crop plant and the question of how to effectively increase maize production and improve other key agronomic traits (nitrogen use efficiency, disease resistance, etc.) remains a major challenge in functional genomics and modern agriculture. The genome of the maize inbred line B73 have been sequenced, assembled, and annotated; however, the large size and complexity of the maize genome make it difficult to identify the critical genes responsible for agriculturally important traits. Comparative analysis of the genomes of maize and other plant species, including *Arabidopsis thaliana* and *Oryza sativa*, can provide a powerful way to identify evolutionarily conserved functional elements and to transfer knowledge from well-studied model organisms to crop plants [1].

Members of the bHLH superfamily of eukaryotic transcription factors act as important transcriptional regulators in many organisms. In these transcription factors, the conserved bHLH domain contains a basic region and a HLH region. The basic region is generally located at the N-terminus of the bHLH domain and binds to a consensus hexanucleotide E-box (CANNTG) [2]. The HLH region is located at the C-terminus of the bHLH domain, contains about 50 amino acids, and is composed of two alpha helixes separated by a loop of variable length. Generally, the HLH region mediates interactions with other bHLH proteins to form homodimeric or heterodimeric complexes [3].

The identification and characterization of the bHLH transcription factors was first reported in mammals and then bHLH proteins were discovered gradually in other eukaryotic species. Previous research on animals has classified the bHLH transcription factors into six subgroups (A–F), based on their protein sequences, differences in the bHLH domains, and comparisons of the functions of the different family members [4]. In animals, bHLH family proteins like BMAL1 (BRAIN AND MUSCLE ARNT-LIKE PROTEIN), CLOCK, HIF1 (HYPOXIA-INDUCIBLE FACTORS) and c-Myc are important signal transduction components involved multiple cellular processes including the circadian clock, cell metabolism, cancer, and disease responses [5].

Genome-wide comprehensive and systematic analyses of bHLH proteins in plants have been carried out in *Arabidopsis*, rice (*Oryza sativa*), poplar (*Populus* sp.), and other species based on genome sequences [1]. In plants, bHLH family proteins such as MYCs, BEEs, PRE (PACLOBUTRAZOL RESISTANCE), BIM (BES1-INTERACTING MYC-LIKE), and PIFs (PHYTOCHROME INTERACTING FACTORS) have been shown to function in multiple cellular processes, including seed germination, shade avoidance response, flowering time regulation, and stress responses [2, 6]. In maize, the first bHLH family member identified was the *R* gene, which is essential for anthocyanins synthesis [7]. However, only a few bHLH proteins have been functionally characterized in maize. Here, to characterize this gene family in maize, we conducted a genome-wide analysis of genes predicted to encode bHLH transcription factors, including gene identification, subfamily classification, phylogenetic analysis, and examination of conserved motifs, exon-intron structure, chromosome distribution, and conserved *cis*-elements in the promoter regions. We further investigated the expression of each bHLH family protein in multiple developmental stages of roots, leaves, and seeds. Thus, this study provides a useful resource for further characterization of the physiological functions of the bHLH transcription factors in maize and other plant species.

## Methods

### Identification of bHLH family proteins in maize

The protein sequences of each bHLH family protein of *Arabidopsis thaliana* were retrieved from the plant transcription factor database (http://planttfdb.cbi.pku.edu.cn) [8]. The protein sequences of the *Zea mays* inbred line B73 were obtained from MaizeGDB (http://www.maizegdb.org, AGPV3) and used to construct a local protein database. BLASTP was used to identify proteins containing bHLH domains from the 63,241 full-length proteins of maize inbred line B73 using the *Arabidopsis* bHLH protein sequences as query sequences. The HMMER program (http://hmmer.janelia.org) was used to identify bHLH proteins in the local protein database with the newest Hidden Markov Model of the bHLH domain (PF00010), which was downloaded from the Pfam database (http://pfam.xfam.org). Redundant protein sequences were removed with program ElimeDupes (https://hcv.lanl.gov/content/sequence/ELIMDUPES/elimdupes.html), or manually by searching in the NCBI database (https://www.ncbi.nlm.nih.gov) and the SMART database (http://smart.embl-heidelberg.de).

### Gene structure and protein motif analysis

Gene Structure Display Server (http://gsds.cbi.pku.edu.cn) was used to analyze the exon–intron structures within the coding sequences and the genomic sequences of each predicted *ZmbHLH*. MEME was used to analyze the conserved protein motifs of maize bHLH proteins using the full-length protein sequences of each putative ZmbHLH family member.

### Multiple alignments and phylogenetic analysis

ClustalX2.1 was used to perform multiple alignments of conserved bHLH domains. A phylogenetic tree was established by the neighbor-joining method using a bootstrap test with 1,000 replications in Phylip (http://evolution.gs.washington.edu/phylip.html). The phylogenetic tree and the original tree were visualized in MEGA5.0 and PolyDraw, respectively.

### Chromosomal locations and gene duplication analysis

The chromosomal positions of *ZmbHLH* loci were obtained from the MaizeGDB. The gene duplication events were defined by three criteria: (1) the covered alignment length was > 80% of the length of the longer gene; (2) the identity of the aligned region was > 80%; the tightly linked genes were involved in only one duplication event.

### Promoter sequence analysis of *ZmbHLH*

The conserved *cis*-elements in the promoter regions of *ZmbHLH* were investigated by downloading the 1500 bp upstream flanking sequences from the transcription start site (TSS) of each putative *ZmbHLH* from Phytozome (https://phytozome.jgi.doe.gov). Promoter sequence analyses were then performed in PlantCARE [9].

### Construction of protein interaction networks

The functional protein–protein interaction networks were generated with STRING (https://string-db.org, Ver10.5) using the protein sequences of the ZmbHLH family to search the maize and *Arabidopsis* databases. All of the sequences of putative ZmbHLH proteins were uploaded into the Multiple Sequences column, and then *Arabidopsis thaliana* was selected to perform the comparison analysis. After BLAST analysis, the highest scoring proteins were used to generate the functional protein interaction network.

### RNA-sequencing (RNA-seq) and gene expression analyses of bHLH transcription factors

RNA-seq data for the *ZmbHLH* genes were obtained from previous studies of differential gene expression in various development stages, organs, and tissues [10–17]. RNA-seq data for each *ZmbHLH* were extracted, analyzed, normalized (the maximum expression value of each *ZmbHLH* gene was set as 1, and then the expression value of this gene in other tissues or conditions was normalized to the maximum expression value), and displayed in heat maps.

### Plant materials and growth conditions

The maize inbred line B73 was used for the experiments in this study. All the plant materials were grown in the greenhouse with a cycle of 12-h light (28 °C) and 12-h darkness (22 °C) and a relative humidity of 50%. All the materials used in the assay were the third leaves of maize seedlings at the three-leaf stage. The base and the tip of the leaves were used to analyze the proximodorsal development of the leaf. All sample materials were collected, immediately frozen in liquid nitrogen, and stored at − 80 °C.

### Total RNA isolation and expression confirmation by RT-qPCR

In our study, total RNA was extracted from plant tissue by using the Ultrapure RNA kit (CWBIO) according to the manufacturer’s instructions. The procedure for RT-qPCR was described in previous studies [18]. The maize *Actin* (GenBank Accession: J01238) gene was used as internal control for normalizing the transcript levels. The primers used are listed in Additional file 1: Table S1.

### Yeast two-hybrid assay

The full-length cDNAs for *ZmbHLH23*, *ZmbHLH61*, *ZmbHLH114*, *ZmbHLH163*, and *ZmbHLH180* were generated by PCR amplifying cDNA from the maize inbred line B73. All the primers used are listed in Additional file 1: Table S1. The amplified full-length fragments of *ZmbHLH23*, *ZmbHLH114*, and *ZmbHLH180* were ligated into the pGADT7 and pGBKT7 vectors (Clontech). *ZmbHLH61* was ligated into the pGBKT7 vector. *ZmbHLH163* was ligated into the pGADT7 vector. The yeast two-hybrid assay was performed according to the manufacturer’s instructions (Clontech).

## Results

### Identification and classification of bHLH family proteins in maize

Examination of the maize genome sequence identified 208 ZmbHLH family proteins in the maize inbred line B73 (Additional file 2: Table S2). This indicates that maize has more bHLH transcription factor family members than other plant species (166 bHLH family members in *Arabidopsis*, 166 in rice, 159 in tomato) [19–21]. Further, ZmbHLH family proteins were classified into 18 subfamilies according to multiple alignments and phylogenetic analysis, and the classification of bHLH family proteins in *Arabidopsis* and rice (Additional file 3 and Additional file 4: Figure S1). The largest subfamily in maize was subfamily XI with 22 members, and subfamily III was the smallest, with only 3 members.

ZmbHLH subfamily XIV (15 members) was closely related to *Arabidopsis* group VII, which includes the PIF subfamily. The recently identified ZmPIFs family members including ZmPIF3.1 (ZmbHLH76), ZmPIF3.2 (ZmbHLH165), ZmPIF4 (ZmbHLH16), and ZmPIF5 (ZmbHLH27) [18, 22, 23] were all classified into this subfamily. Consistent with this, all seven members of subfamily XIV (ZmbHLH 16, 27, 36, 76, 115, 165 and 198, Additional file 3) contain conserved Active Phytochrome A (APA) or Active Phytochrome B (APB) domains, which responsible for physically interact with the photoreceptors phyA or phyB, respectively [23].

Maize subfamily XVII (16 members) was grouped closely with *Arabidopsis* group X*.* As previous reported, the members of subfamily X in *Arabidopsis* have incomplete or mutated basic regions, which may interfere with their ability to bind to DNA but does not affect the formation of dimers with other bHLH proteins. Therefore, these atypical bHLH transcription factors may negatively regulate the molecular functions of other bHLH transcription factors by forming heterodimers [2]. Therefore, we speculate that the ZmbHLH proteins in subfamily XVII might have similar molecular functions.

### Analysis of protein structures

MEME analysis of the ZmbHLH family proteins identified 10 putative conserved protein motifs (Motifs 1–10, Additional file 3). The conserved bHLH domain was predicted in Motif 1 (containing the basic region and the first helix) and Motif 2 (second helix); since the protein sequences of the loop region between helix 1 and helix 2 are less conserved. Most of the ZmbHLH family proteins contained Motif 1 and Motif 2 (Additional file 3), while ZmbHLH8, ZmbHLH31, ZmbHLH45, ZmbHLH126, ZmbHLH138, ZmbHLH191, and ZmbHLH197 have only Motif 1, and ZmbHLH136 and ZmbHLH137 have only Motif 2. In each subfamily, the components of the conserved motifs for most of the proteins were similar (Additional file 3). For example, Motifs 1, 2, and 3 were identified in all 22 members of subfamily XI, and Motifs 1, 2, and 4 were identified in 19 of the 20 members of subfamily X. In subfamily XV, Motifs 1, 2, and 6 were identified in 12 of the 13 members, with ZmbHLH85 lacking only Motif 6.

### Conserved amino acid residues in the bHLH domain

**Fig. 1:**
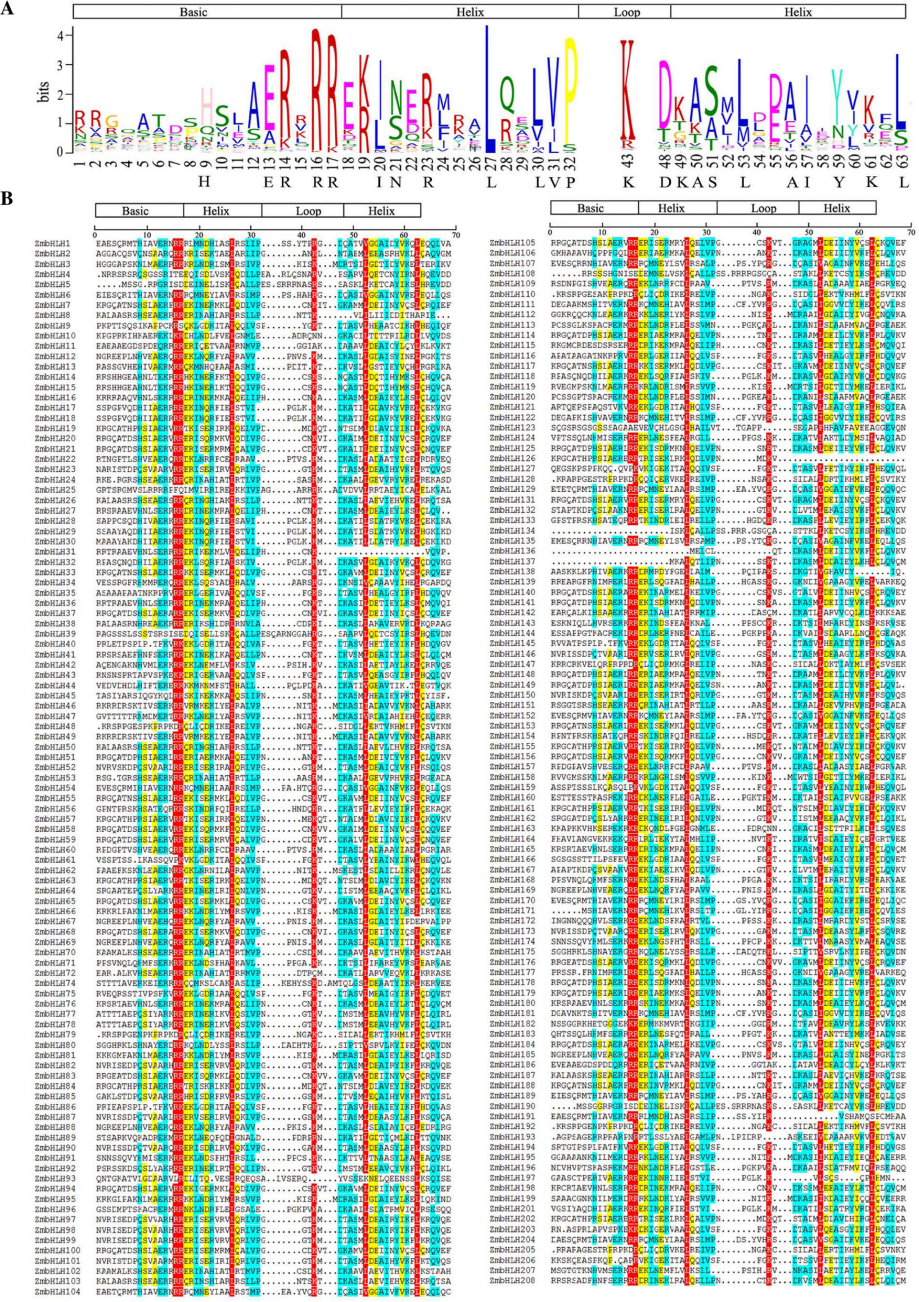
Multiple alignments of the bHLH domains in the ZmbHLH family proteins. **a** Conserved amino acid analysis of ZmbHLH domains. The height of each amino acid indicates the conservation ratio. The amino acids with a conservation ratio more than 50% are indicated in black letters. **b** Multiple alignments of ZmbHLH domains. Amino acids with greater than 75% identity are shown in red, those with 50 to 75% identity are shown in cyan, and those with 33 to 50% identity are shown in yellow. Dotted lines indicate gaps

Analysis of conserved amino acid residues in the bHLH domain using multiple alignments of only the bHLH domain of each putative ZmbHLH protein showed four conserved regions, including the basic region, two helixes, and a loop region (Fig. 1a). The basic region and two helixes were highly conserved in most of ZmbHLH family proteins, except the basic region was not present in ZmbHLH134, ZmbHLH136, and ZmbHLH137, and the second helix region was not present in ZmbHLH31 and ZmbHLH126 (Fig. 1b). The loop region between the two helixes, however, showed significant variation in the number and sequence of the amino acids. In most of ZmbHLH family proteins, the loops region generally consisted of 6–15 amino acids (AA), but only three ZmbHLH proteins had much longer loop regions (30 AA for ZmbHLH73, 23 AA for ZmbHLH130, and 41 AA for ZmbHLH200), which suggest these three members might have distinct regulatory mechanism.

Multiple sequence alignment showed that 23 amino acid residues were highly conserved (> 50% consensus ratio), and that six of those were conserved with a > 75% consensus ratio (Fig. 1b). As expected, all of these conserved amino acid residues are consistent with previous studies of the bHLH domain in other plant species [2]. Among these 23 conserved residues, some are highly conserved in the basic regions (His-9, Glu-13, Arg-14, Arg-16, and Arg-17), the first helix region (Ile-20, Asn-21, Arg-23, Leu-27, Leu-30, Val-31, and Pro-32), the loop (Lys-43 and Asp-48), or in the second helix region (Lys-49, Ala-50, Ser-51, Leu-53, Ala-56, Ile-57, Tyr-59, Lys-61, and Leu-63).

### Analysis of gene structures

Gene structure analysis can help reveal the evolutionary relationships of the members of gene families. Analysis of the intron–exon composition of each member revealed that 30 *ZmbHLH* genes have only exons, 152 *ZmbHLH* genes have two to seven exons, and six *ZmbHLH* genes have more than ten exons (Additional file 3). As expected, gene structure analysis revealed that most members in the same subfamily had similar intron–exon compositions, including the numbers and length of exons. For example, all the members of subfamily XI have more than five exons, and all the members of subfamily XV have only one exon. Taken together, our results indicate that ZmbHLH transcription factors in the same subfamily had similar gene structures and contained similar conserved protein motifs, further suggesting that the members in the same subfamily have close evolutionary relationships.

### Chromosome distribution and gene duplication

**Fig. 2:**
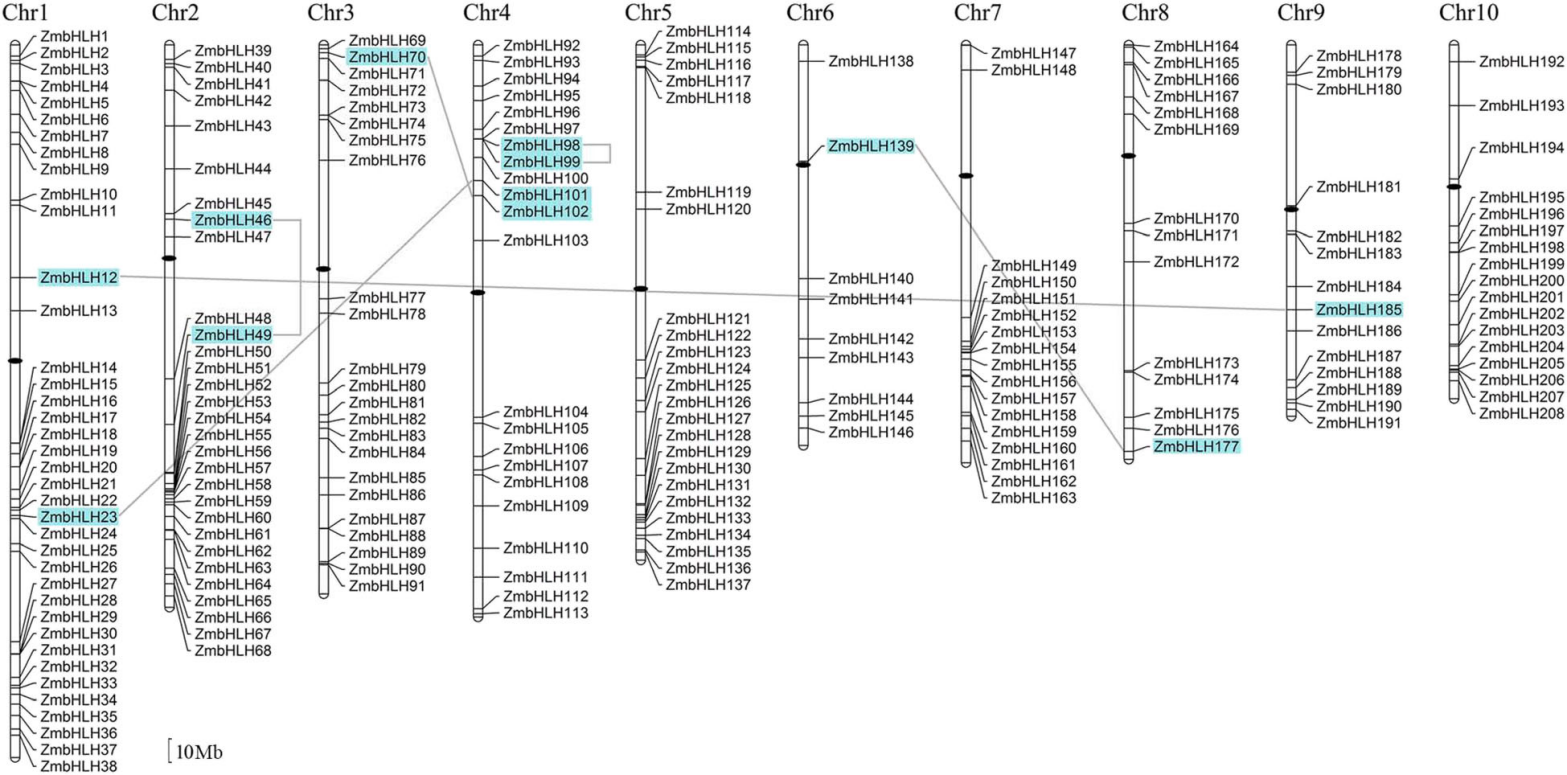
Chromosome distribution and duplication events for *ZmbHLH* genes. The position of each *ZmbHLH* is noted on the right side of each chromosome (Chr). Centromeres are indicated by black ovals. Gene names highlighted with cyan shadows and linked by black lines are the products of gene duplication. Scale bar, 10 Mb

Amplification of gene families by duplication can result in some chromosomes having many loci from the same gene family. To examine their chromosomal distribution, each *ZmbHLH* gene was mapped to a physical position on the maize chromosomes, according to the gene annotation information. This showed that the *ZmbHLH* genes were non-randomly located on the ten maize chromosomes (Fig. 2). Most of the *ZmbHLH* genes were located on the long arms of Chr1 (25 members), Chr2 (21 members), Chr7 (15 members), and Chr10 (14 members); 17 members were located on the short arm of Chr5. More than one-third of the *ZmbHLH* genes were located on Chr1 (38 members) and Chr2 (30 members). Only nine *ZmbHLH* genes were found on Chr6. *ZmbHLH139* and *ZmbHLH181* mapped near the centromeres of Chr6 and Chr9, respectively, and *ZmbHLH147* and *ZmbHLH164* mapped at the ends of Chr7 and Chr8, respectively.

Gene duplication plays an important role in gene amplification. Twelve duplicated *ZmbHLH* genes with genomic sequence identity greater than 80% were identified in the maize genome. The pairs of duplicated genes were *ZmbHLH12* and *ZmHLH185*, *ZmbHLH23* and *ZmbHLH101*, *ZmbHLH46* and *ZmbHLH49*, *ZmbHLH70* and *ZmbHLH102*, *ZmbHLH98* and *ZmbHLH99*, and *ZmbHLH139* and *ZmbHLH177* (Fig. 2). The intron–exon structures were also similar between the two members in each pair of duplicated genes, suggesting that gene duplication events played a significant role in bHLH-domain gene amplification in the maize genome.

### Conserved *cis*-elements in the promoter regions of *ZmbHLH* genes

In addition to diverging in function, members of gene families can diverge in expression pattern. To examine potential differences in gene regulation, we looked for *cis*-elements in the promoter regions of the various *ZmbHLH* genes. PlantCARE promoter sequence analysis identified multiple *cis*-elements known to be involved in development and stress responses in the promoter regions (1500 bp upstream of the transcriptional start sites, TSS) of *ZmbHLH* sequences, which were extracted from MaizeGDB. The most commonly predicted *cis*-elements in the promoter regions of various *ZmbHLH* genes were elements related to the light response which indicated that light signals might play important roles in the transcriptional regulation of *ZmbHLH* genes (Additional file 5: Table S3). For example, 188 (90.38%) *ZmbHLH* genes and 187 (89.90%) *ZmbHLH* genes contain the G-box *cis*-elements.

*Cis*-elements related to other plant responses were also identified in the promoter regions of various *ZmbHLH* genes, including elements responsive to auxin (AuxRR-core), salicylic acid (ARE and TCA-elements), jasmonic acid (CGTCA and TGACG motif), abscisic acid (ABRE element), gibberellins (GARE motif and P-box), heat stress (HSE element), low-temperature (LTR element), and endosperm-specific expression (Skn-1 element) (Additional file 5: Table S3).

### Expression of *ZmbHLH* genes in root development

**Fig. 3:**
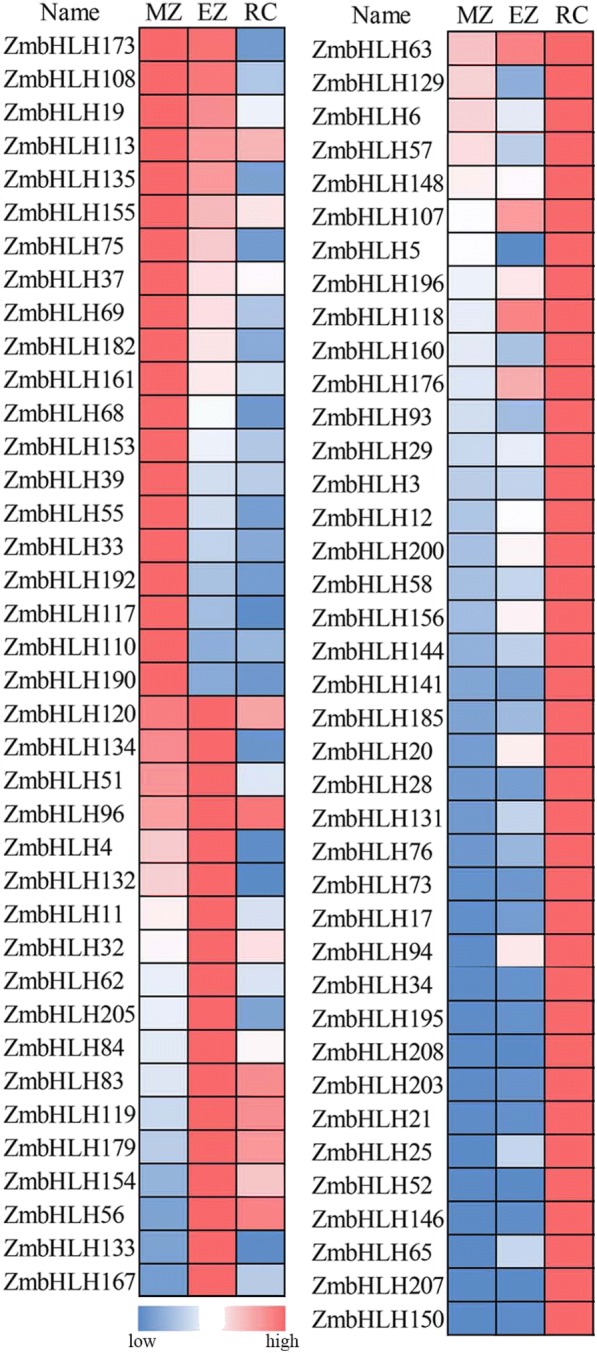
Expression profile of *ZmbHLH* genes in root. Relative expression values of *ZmbHLH* genes in the meristem zone (MZ), elongation zone (EZ), and root cortex (RC), in 5-day-old seedlings of maize inbred line B73. The original expression values of each *ZmbHLH* gene are listed in Additional file 6: Table S4

To investigate the potential roles of *ZmbHLHs* in root development, we used RNA-seq data produced by previous studies [11, 15]. These data showed that *ZmbHLH* genes are highly expressed in the meristem zone (20 *ZmbHLHs*), elongation zone (18 *ZmbHLHs*), and root cortex (40 *ZmbHLHs*; Fig. 3) in the primary root of 5-day-old seedlings of maize B73. Interestingly, we noticed that *ZmbHLH4*, *ZmbHLH28*, *ZmbHLH119*, *ZmbHLH144* and *ZmbHLH207* are extremely high expressed in the different root parts, based on the original FPKM values (FPKM> 100) (Additional file 6: Table S4). *ZmbHLH4* and *ZmbHLH51* are extremely high expressed in the elongation zone, which consistent with their functions in cell wall biogenesis [24] and root hair production [11, 25]. *ZmbHLH28*, *ZmbHLH144*, and *ZmbHLH207* are highly expressed in the root cortex, and *ZmbHLH119* is highly expressed in the elongation zone, suggesting they might also involve in root development. In Fig. 3, among the 78 genes that have a relatively high expression levels in the roots, 34 genes contain the auxin-responsive element, 39 contain the meristem-specific element, and 48 contain the gibberellin-responsive element. Additionally, we also identified that 84 *ZmbHLH* genes are not expressed in the root tissue (FPKM values < 1; Additional file 6: Table S4).

### Expression of *ZmbHLH* genes in leaf development

**Fig. 4:**
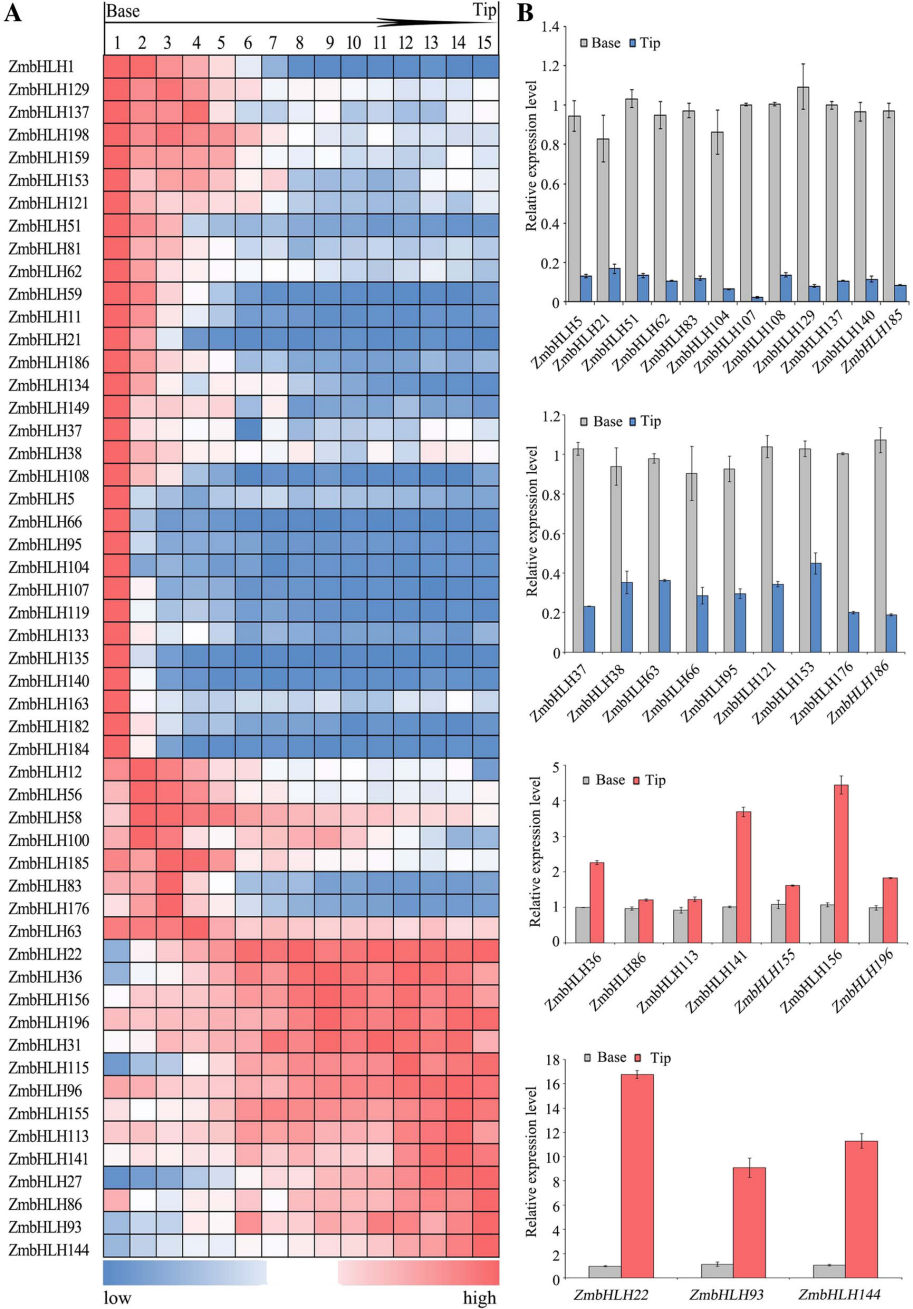
Expression profile of *ZmbHLH* genes in the leaves. **a** Relative expression values of *ZmbHLH* genes in the different regions of the third leaves of 9-day-old seedlings of maize inbred line B73. The original expression values of each *ZmbHLH* gene are listed in Additional file 7: Table S5. **b** RT-qPCR analysis of *ZmbHLH* genes involved in leaf development

To investigate the potential roles of *ZmbHLHs* in leaf development, we collected RNA-seq data from three previous studies [10, 11, 15, 26]. In the previous studies, to gather a gradient gene expression analysis from immature to mature leaf tissue, the full-expanded third leaf of the seeding plants of maize B73 was cut into 4 or 10 parts, or 15 slices (M1–M15) from the base to the tip [10, 11, 15, 26]. In the immature tissue of the basal part of the leaf, 39 *ZmbHLH* genes including *ZmbHLH1*, *ZmbHLH129*, and *ZmbHLH137*, were highly expressed, and their expression levels gradually decreased from the base to the mature tissue at the tip of the leaf (Fig. 4a, Additional file 7: Table S5). As shown in the Fig. 4b, these genes that involved in proximal-distal axis leaf development have been verified by RT-qPCR. We speculate that these genes with high expression levels in the base region of the leaf may be involved in cell division and elongation. Consistently, RSL4 (ROOT HAIR DEFECTIVE 6-LIKE 4), the homolog of ZmbHLH186 in *Arabidopsis*, has been identified play an important role in control cell growth and size [27].Additionally, we found that most of the promoters of genes with high expression in the basal region of leaf contain auxin-responsive elements, gibberellin-responsive elements, also suggesting that they may play an essential role in the cell division and elongation in the early leaf development (Additional file 7: Table S5).

In contrast, we also identified that 14 *ZmbHLH* genes including *ZmbHLH22*, *ZmbHLH36*, and *ZmbHLH156*, were highly expressed at the tip of the leaf and decreased in expression down to the base of the leaf (Fig. 4a). RT-qPCR results showed that the expression of *ZmbHLH22*, *ZmbHLH41, ZmbHLH93*, *ZmbHLH144, and ZmbHLH156* were significantly higher at the tip than at the base and consistent with the previous RNA-seq data (Fig. 4b; Additional file 7: Table S5). We also found that multiple *bHLH* genes including *ZmbHLH96*, *ZmbHLH113*, and *ZmbHLH144* are extremely high expressed (RPKM> 50) in the mature region of leaf which indicate they may associate with the photosynthesis (Additional file 7: Table S5a). Consistently, ZmbHLH113 has been identified play essential roles in photosynthesis through control the transcription of *NADP-ME* (*NADP-Malic Enzyme*) [28]. Interestingly, we identified that the promoter regions of all of these genes contain at least one element related to light responses (G-box, GAG-motif, GTGGC-motif and so on). Moreover, 12 genes contain *cis*-acting regulatory elements involved in the response to methyl jasmonate (TGACG-motif and CGTCA-motif) and 5 genes have an HSE element involved in heat stress responses. Consistently, *MYC2*, *MYC3*, and *MYC4,* the homolog of *ZmbHLH22* in *Arabidopsis*, encode JAZ-interacting bHLH type transcription factors and have been demonstrated play important roles in jasmonate responses [29]. Therefore, based on the above RNA-seq data analysis and experimental verification, we believe that those genes highly expressed at the tip region of leaves might function in photosynthesis, defense or stress responses.

**Fig. 5:**
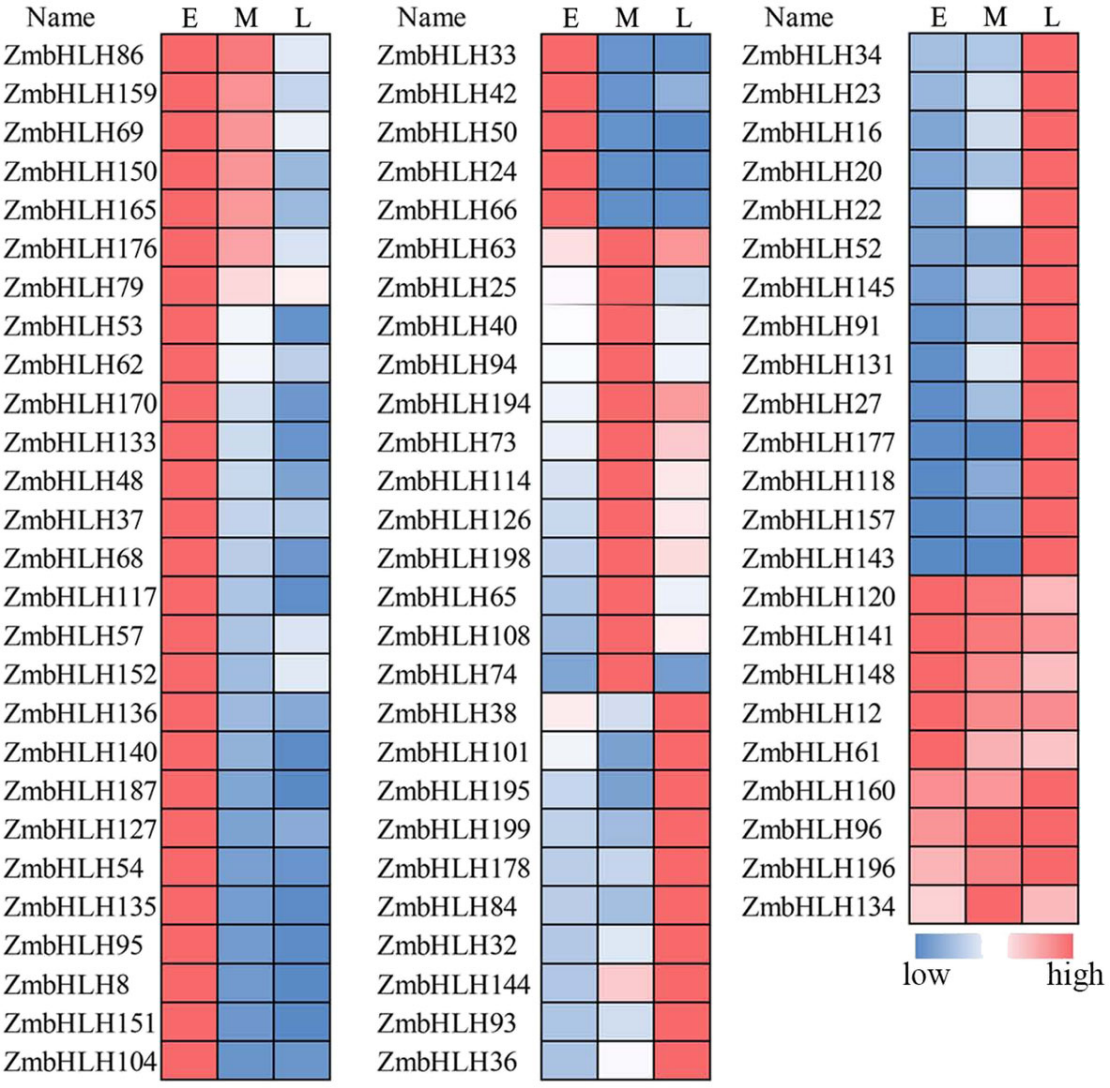
Expression profile of *ZmbHLH* genes in the different developmental stages of the leaf auricle. Relative expression value of each *ZmbHLH* genes in the early (E), middle (M), and late (L) development stages of the leaf auricle of maize inbred line B73. The original expression values of each *ZmbHLH* gene are listed in Additional file 8: Table S6

Leaf angle is an important agricultural trait that affects planting density and grain yield and is affected by auricle development in the ligule region [16]. Regression analysis performed on RNA-seq data from early, middle, and late stages of auricle development [16] identified that 32 *ZmbHLH* genes are highly expressed in the early stages of auricle development (Fig. 5). Some of these *bHLH* genes, including *MALE STERILITY32* (*MS32*, *ZmbHLH62*) have been identified as acting in both cell division and differentiation, and also highly expressed in the base region of leaf (Fig. 4a) [30]. This suggests that these genes might also act in cell division or differentiation in the leaf auricle.

Twelve *ZmbHLH* genes were highly expressed in the middle stage of auricle development, and 24 were highly expressed in the late stage of auricle development (Fig. 5). Also 9 *ZmbHLH* genes were highly expressed at all stages of auricle development. Among these, *ZmbHLH12* is the most highly expressed at all stages of auricle development (RPKM> 190; Additional file 8: Table S6). The homologs of *ZmbHLH4*, *ZmbHLH5*, *ZmbHLH39*, and *ZmbHLH108* have been identified controlling the angle of leaves in rice [31], which suggest that they may have similar functions in maize. All these analyses indicated that ZmbHLH transcription factors might be involved in development of the auricle and the regulation of leaf angle.

### Expression of *ZmbHLH* genes in seed development

**Fig. 6:**
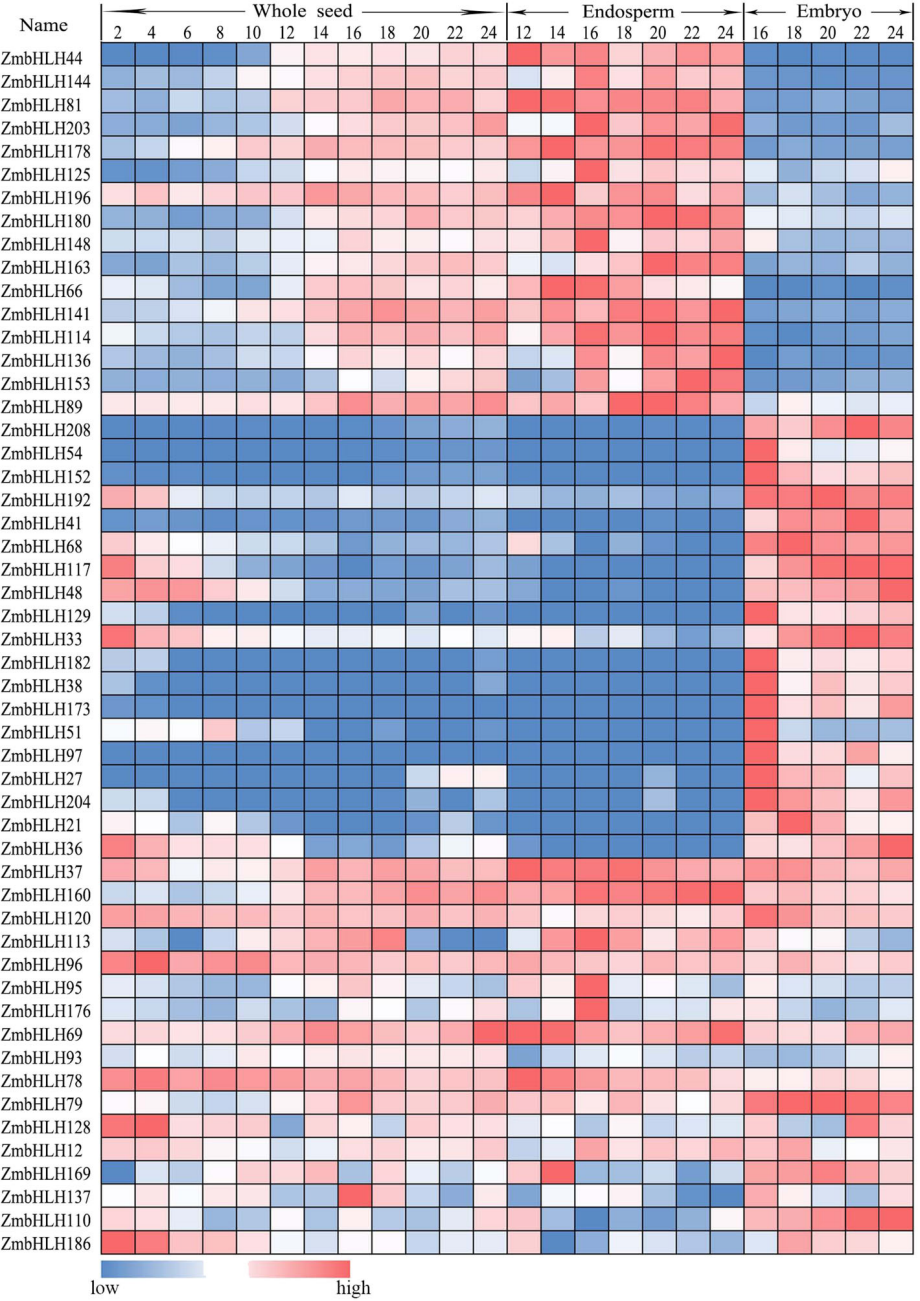
Expression of *ZmbHLH* genes in seed development. Relative expression value of each *ZmbHLH* gene across developmental stages of whole seed, endosperm, and embryo. Numbers at the top of each column indicate the number of days after pollination. The original expression values of each *ZmbHLH* gene are listed in Additional file 9: Table S7

To investigate the potential roles of *ZmbHLHs* in seed development, RNA-seq data from the different cell types of whole seed, embryo, and endosperm at different days after pollination were collected and analyzed [11–15]. There are 157 *ZmbHLH* genes expressed during seed development (FPKM > 1) (Additional file 9: Table S7a). Among the 52 highly expressed seed development related genes (FPKM > 10), 16 *ZmbHLH* genes (including *ZmbHLH44* and *ZmbHLH144*) are very highly expressed in the endosperm, 19 *ZmbHLH* genes (including *ZmbHLH208* and *ZmbHLH54*) are especially highly expressed in the embryo (Fig. 6). Consistently, *ZmbHLH44* (*OPAQUE11*) has been demonstrated control the storage nutrient metabolism in maize endosperm [32].

There are also 17 *ZmbHLH* genes (including *ZmbHLH37* and *ZmbHLH160*) highly expressed in both endosperm and embryo development, which may be involved in the development of the entire seed (Fig. 6; Additional file 9: Table S7). Interestingly, there are two genes (including *ZmbHLH87* and *ZmbHLH185*) are specifically expressed at the transfer cell (Additional file 9: Table S7c), which suggest that they may regulate the process of the grain filling thus crucial for seed production.

### Protein interaction networks

**Fig. 7:**
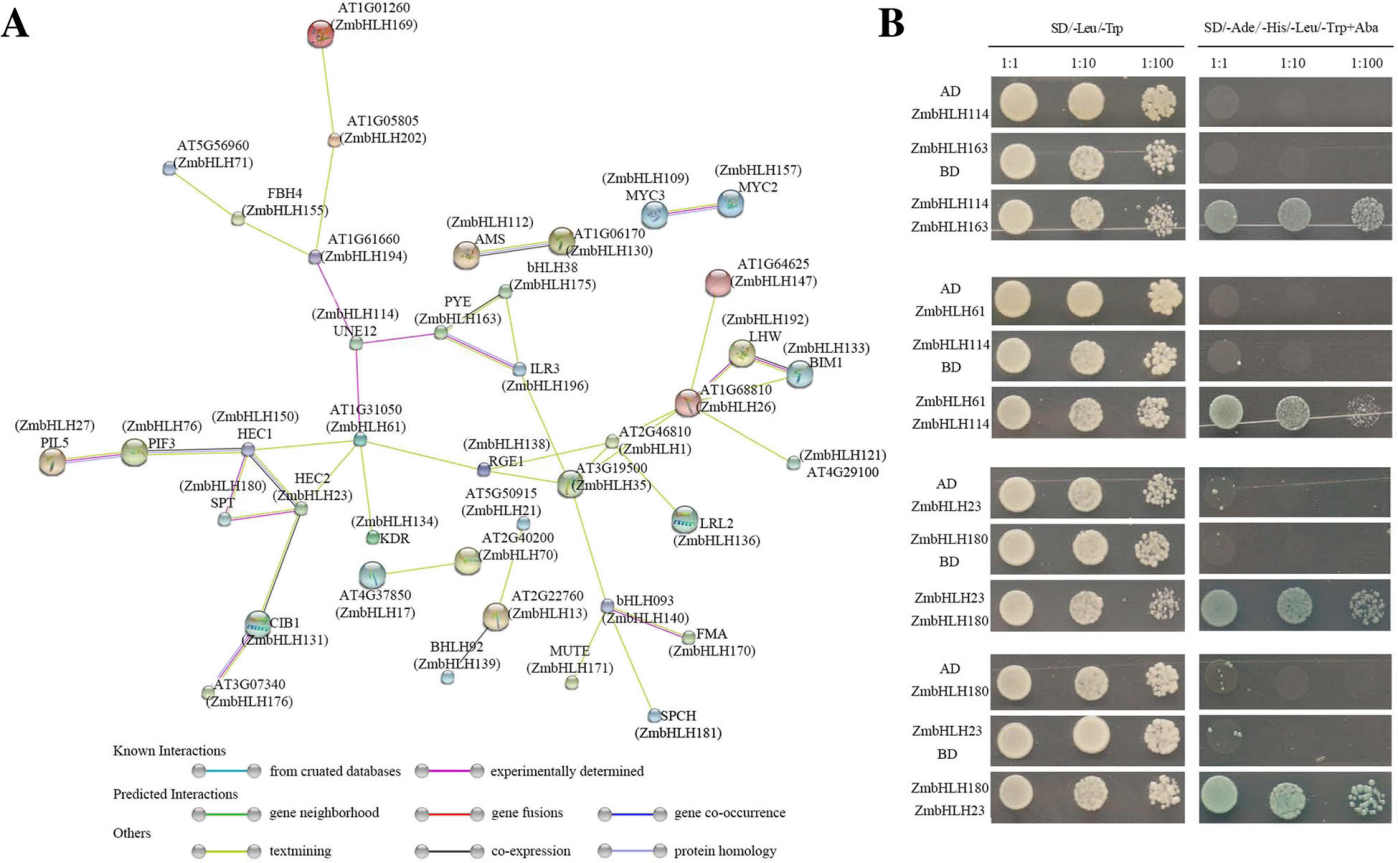
Functional protein association network and Yeast two-hybrid assay. **a** Protein interaction network analysis revealed that most ZmbHLH proteins interact with more than one other bHLH protein. Line colors indicate the different kinds of evidence used to predict the network of protein interactions. **b** Yeast two-hybrid analysis of protein–protein interactions of various ZmbHLH proteins. AD and BD represent empty pGADT7 and pGBKT7 vectors, respectively. SD/-Leu/-Trp represents the synthetic dextrose media (SD) lacking Leu and Trp. SD/-Ade/-His/-Leu/-Trp/Aba indicates SD medium lacking Ade, His, Leu, and Trp but with Aureobasidin A

Different bHLH proteins can form homodimers or heterodimers thus to bind DNA and regulate the transcription of downstream targets; therefore, protein–protein interactions are fundamentally important to bHLH protein function. Analysis of protein interaction networks (see Methods) revealed that most of the ZmbHLH proteins interacted with more than one bHLH protein. In particular, seven ZmbHLH proteins (ZmbHLH150, ZmbHLH23, ZmbHLH1, ZmbHLH26, ZmbHLH 119, ZmbHLH35, and ZmbHLH61) could interact with four or more other bHLH proteins (Fig. 7a). Additional file 10: Table S8 describes the query sequences and annotated functions of the network proteins detailed. These results suggest that these proteins may work together to regulate plant growth and development, although more research is needed.

To verify the predicted protein interaction networks, we selected several proteins that interacted with more than one bHLH protein and conducted yeast two-hybrid experiments. To this end, the full-length cDNA fragments of different members were ligated into the pGADT7 and pGBKT7 vectors. Yeast two-hybrid assays demonstrated that ZmbHLH114 can interact with ZmbHLH61, and ZmbHLH163 in vitro, confirming the predicted interactions identified above (Fig. 7b). Additionally, assays also verified the predicted interaction between ZmbHLH23 and ZmbHLH180.

Indeed, our expression analysis showed that ZmbHLH180 is highly expressed in roots, auricle and seed, but its partner ZmbHLH23 is expressed in auricle, indicating that ZmbHLH23 and ZmbHLH180 may act together in auricle. By contrast, ZmbHLH114 is expressed in all analyzed RNA-seq data (including roots, leaves, auricle and seed), so the spatial and temporal interaction between ZmbHLH114 and other partners depends on the expression site of the interacting gene. The physical interactions of these various kinds of ZmbHLH proteins suggest that they may function in different manners by forming various kinds of homodimers or heterodimers at different tissues, organs, or stages thus to coordinately regulate different cellular processes.

## Discussion

Transcription factors play important roles in the regulation of downstream targets, making them crucial to diverse biological processes in plant growth, development, and stress responses. Recent studies have revealed that 39,469 protein-coding genes exist in the maize genome, 2289 of which encode transcription factors in 56 families [8]. However, only a few of the transcription factor families have been systematically studied in maize, such as the MYB [33], bZIP [34], WRKY [35], and NAC [36] families. The bHLH transcription factors comprise a large family in higher plants, and numerous studies have shown that bHLH-type transcription factors are involved in diverse biological processes in plant growth, development, and stress responses [2]. However, we lack a systematic characterization of bHLH genes in maize. From the PlantTFDB website, we found that higher plants (including *Brassica napus*, *Panicum virgatum*, and *Glycine max*) have a large number of bHLH family genes, but lower plants and fungi (including *Bathycoccus*, *Helicosporidium*, *Ostreococcus lucimarinus*, *Ostreococcus tauri*, and *Picochlorum sp. SENEW3*) each have only one bHLH family gene. This indicates that this large gene family has undergone substantial expansion, with potential subfunctionalization and neofunctionalization, as well as changes in gene expression patterns and protein–protein interactions. In this study, we identified 208 bHLH family proteins in maize through a genome-wide analysis (Additional file 3). Some of these proteins have been previously studied. For example, maize BA1 (ZmbHLH85) participates in the regulation of shoot architecture [37], MS23 (ZmbHLH164) and MS32 (ZmbHLH62) are necessary for plant reproduction, and MS23 is the earliest acting factor to control the tapetal differentiation [30, 38]. ZmKS (ZmbHLH84) is involved in abscisic acid responses [39]. All these evidences suggest that these different bHLH family proteins play important roles in various biological processes.

Based on phylogenetic analysis, we classified the ZmbHLH family proteins into 18 subfamilies (Additional file 3). Protein motif and gene structure analysis showed that different members of the same subfamily likely share evolutionary origins and might have similar physiological functions. For example, ZmbHLH subfamily XIV is closely related to group VII of the PIF family in *Arabidopsis*, which play important roles in light signal transduction, seed germination, shade avoidance, and photomorphogenesis [6]. In maize, ZmPIF3.1 (ZmbHLH76) and ZmPIF3.2 (ZmbHLH165) interact with ZmphyB1 in light signal transduction [23]. Additionally, ZmPIF4 (ZmbHLH16) and ZmPIF5 (ZmbHLH27) share similar functions with AtPIF4 and AtPIF5, since they can partially rescue the reduced hypocotyl elongation of *Arabidopsis pif1 pif3 pif4 pif5* quadruple mutants [18].

To further explore the physiological role of the bHLH family in plant growth and development, we systematically analyzed the expression profiles of *ZmbHLH* family genes in different organs including root, leaf blade, auricle, and seed development. Generally, genes related to cell division are highly expressed in young tissue and genes involved in cell elongation and expansion are highly expressed in the mature tissues [26]. For example, *ZmbHLH37*, *ZmbHLH62*, and *ZmbHLH135* were highly expressed in young tissues of the root, leaf, and auricle, which suggest that these three genes might be involved in the regulation of cell division. Thorough comparison of the expression profiles of *ZmbHLH* genes in different organs and specific developmental stages provides key information for further functional characterization. Analysis of conserved *cis*-elements in the promoter regions of different *ZmbHLH* genes identified multiple *cis*-elements involved in plant development and stress responses (Additional file 5: Table S3). The promoter regions of most of the genes that are highly expressed in young tissues contain auxin-responsive, meristem expression, and the gibberellin-responsive elements. By contrast, the promoter regions of most of the genes specifically expressed in mature tissues contain *cis*-elements involved in the stress response, and defense responses. The promoter regions of genes highly expressed in the mature leaves also contain multiple light-responsive *cis*-elements. The promoter regions of the most of *ZmbHLH* genes related to seed development contain the endosperm expression-related elements. Based on the analysis of these *cis*-acting elements and specific gene expression patterns of different *ZmbHLH* family members, we can identify conserved regulatory modules for these different members in specific tissues or developmental stages. In addition, predicted protein interaction networks provide a theoretical basis for studying the physiological functions of different bHLH family proteins. The physical interaction of different ZmbHLH family proteins suggests that they might function in various cellular processes by forming different kinds of homodimers or heterodimers at different tissues or organs, or development stages.

## Conclusion

In this study, 208 bHLH transcription factors of maize inbred B73 were subjected to identification, classification, phylogenetic reconstruction and conserved motif characterization. In addition, the expression patterns of 208 *ZmbHLH* genes in different organs and tissues, combined with the conserved *cis*-elements analysis and protein interaction analysis were also analyzed. This comprehensive and systematic analysis of bHLH transcription factors in maize reveals that they are probably involved in multiple physiological processes in plant growth and development. Finally, our comprehensive and systematic analysis provides fundamental and useful information for studies to further investigate the physiological and molecular function of different bHLH transcription factor family members in maize and other plants.

## Additional files

Additional file 1: **Table S1.** Primers used in this study. (XLSX 127 kb)

Additional file 2: **Table S2.** Annotation information for 208 bHLH family genes in maize, 166 bHLH family genes in rice and 166 bHLH family genes in *Arabidopsis. (XLSX 37 kb)*

Additional file 3: Phylogenetic tree, protein motifs, and gene structure of bHLH family members in maize. **a** Phylogenetic tree of the 208 proteins in the bHLH family in maize. The subfamily classification is indicated by Roman numerals on the left. The bootstrap value is indicated on each branch. **b** Conserved motifs of the ZmbHLH family proteins. Ten putative conserved motifs are indicated by color, and the length of the black line represents the length of each protein sequence. Scale bar, 100 amino acids. **c** Exon–intron analysis of *ZmbHLH* family genes. Line length indicates genomic sequence length. Scale bar, 1 kb. (PNG 605 kb)

Additional file 4: **Figure S1.** Original tree of bHLH family genes in maize, rice and *Arabidopsis.* Radial tree of bHLH domains in maize, rice and *Arabidopsis.* The maize bHLH subfamilies are indicated by Roman numerals. (PDF 242 kb)

Additional file 5: **Table S3.** Distribution and annotation information for the *cis*-elements in the promoter regions of *ZmbHLH* genes. (XLSX 196 kb)

Additional file 6: **Table S4.** Expression of *ZmbHLH* genes in maize root tissue. (XLSX 106 kb)

Additional file 7: **Table S5.** Expression of *ZmbHLH* genes across a developmental gradient of maize leaf tissue. (XLSX 82 kb)

Additional file 8: **Table S6.** Expression of *ZmbHLH* genes at different development stages of maize leaf auricle tissue. (XLSX 18 kb)

Additional file 9: **Table S7.** Expression of *ZmbHLH* genes at different development stages of maize seed tissue. (XLSX 169 kb)

Additional file 10: **Table S8.** A complete list of bHLH proteins in the protein network prediction. (XLSX 12 kb)

## Abbreviations

bHLH: basic helix-loop-helix
FPKM: Fragments per kilobase of transcript per million fragments mapped
RPKM: Reads per kilobase per million mapped reads
TSS: The transcription start site
ZmbHLH: *Zea mays* basic helix-loop-helix
