# Supplementary figures and images for: Genome-wide analysis of the basic Helix-Loop-Helix (bHLH) transcription factor family in maize
Source: BMC Plant Biol. 2018 Oct 16;18:235. doi: 10.1186/s12870-018-1441-z (PMC6192367; doi:10.1186/s12870-018-1441-z)

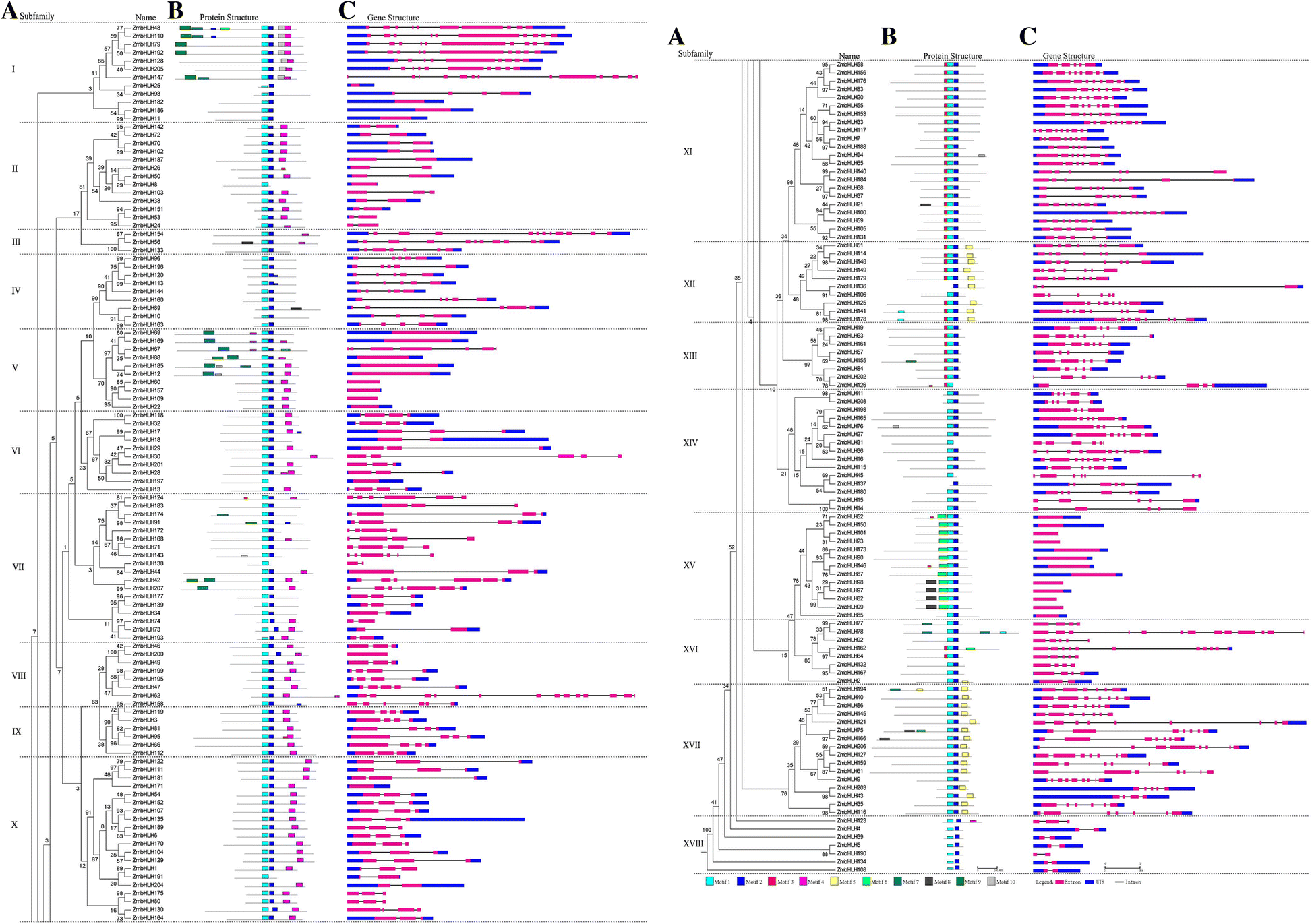

Supplement: Supplementary file 3 — Phylogenetic tree, protein motifs, and gene structure of bHLH family members in maize. a Phylogenetic tree of the 208 proteins in the bHLH family in maize. The subfamily classification is indicated by Roman numerals on the left. The bootstrap value is indicated on each branch. b Conserved motifs of the ZmbHLH family proteins. Ten putative conserved motifs are indicated by color, and the length of the black line represents the length of each protein sequence. Scale bar, 100 amino acids. c Exon–intron analysis of ZmbHLH family genes. Line length indicates genomic sequence length. Scale bar, 1 kb. (PNG 605 kb) [file 12870_2018_1441_MOESM3_ESM.png]

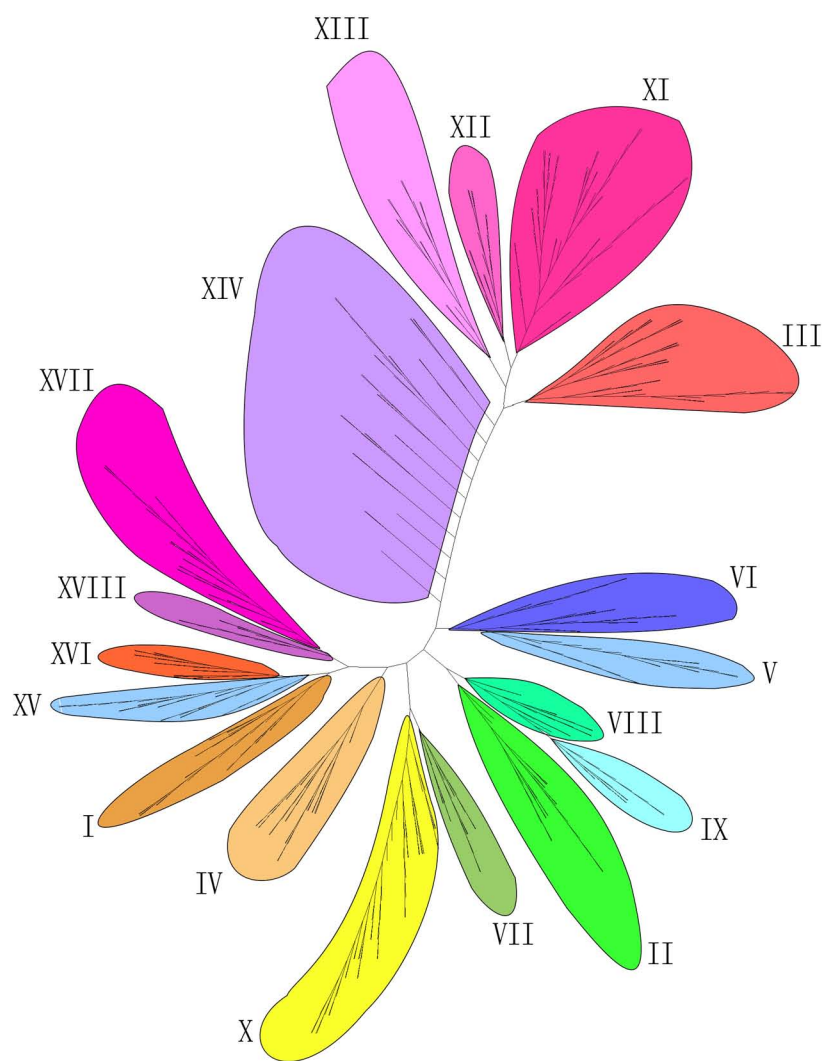

Supplement: Supplementary file 4 — Figure S1. Original tree of bHLH family genes in maize, rice and Arabidopsis. Radial tree of bHLH domains in maize, rice and Arabidopsis. The maize bHLH subfamilies are indicated by Roman numerals. (PDF 242 kb) [file 12870_2018_1441_MOESM4_ESM.pdf]
